# Phosphoproteomics technologies and applications in plant biology research

**DOI:** 10.3389/fpls.2015.00430

**Published:** 2015-06-16

**Authors:** Jinna Li, Cecilia Silva-Sanchez, Tong Zhang, Sixue Chen, Haiying Li

**Affiliations:** ^1^College of Life Sciences, Heilongjiang UniversityHarbin, China; ^2^Proteomics and Mass Spectrometry, Interdisciplinary Center for Biotechnology Research, University of FloridaGainesville, FL, USA; ^3^Plant Molecular and Cellular Biology Program, Department of Biology, UF Genetics Institute, University of FloridaGainesville, FL, USA

**Keywords:** phosphoproteomics, enrichment, quantification, phosphorylation site mapping, plant biology

## Abstract

Protein phosphorylation has long been recognized as an essential mechanism to regulate many important processes of plant life. However, studies on phosphorylation mediated signaling events in plants are challenged with low stoichiometry and dynamic nature of phosphorylated proteins. Significant advances in mass spectrometry based phosphoproteomics have taken place in recent decade, including phosphoprotein/phosphopeptide enrichment, detection and quantification, and phosphorylation site localization. This review describes a variety of separation and enrichment methods for phosphoproteins and phosphopeptides, the applications of technological innovations in plant phosphoproteomics, and highlights significant achievement of phosphoproteomics in the areas of plant signal transduction, growth and development.

## Introduction

Phosphorylation is one of the most important post-translational modifications (PTMs) of proteins (Pawson and Scott, 1997). Approximately one-third of the proteins are modified by phosphorylation (Hubbard and Cohen, 1993). The kinase mediated covalent addition of a phosphate group to serine, threonine, and tyrosine residues in eukaryotes, and other amino acids such as histidine, aspartate, glutamate, lysine, arginine, and cysteine in prokaryotes and the subsequent removal of the phosphate groups by protein phosphatases constitute important signaling and regulatory mechanisms in living organisms (Batalha et al., 2012). Reversible protein phosphorylation regulates a wide range of cellular processes such as transmembrane signaling, intracellular amplification of signals, and cell-cycle control. Protein phosphorylation often leads to protein structural changes that can directly modulate protein activity, and induce changes in interaction partners or subcellular localization (Jørgensen and Linding, 2008). The cascade of protein phosphorylation in a signaling pathway provides the backbone for complex signaling networks and regulatory processes in plant cells, including hormone sensing (Park et al., 2009), and environmental stress responses (Mishra et al., 2006). Thus, the analysis of signaling pathways in plants has often been focused on protein kinases. Traditional studies, however, described the phosphorylation of a single substrate by a particular kinase. Based on genome annotation, protein kinases were found to make up about 5.5% of the Arabidopsis genome (The Arabidopsis Genome Initiative, 2000), which is nearly twice as many as in the human genome (Manning et al., 2002). This indicates high specificity and complex networks of phosphorylation events in plants (Schulze, 2010). Many plant protein kinases have been identified to play essential roles in response to a variety of stresses including salt stress, cold stress, and pathogen invasion. Deciphering the molecular events occurring in stress responses will enhance our understanding of the biological processes in plants (De la Fuente van Bentem et al., 2006; Stecker et al., 2014).

The combination of phosphoprotein/phosphopeptide enrichment techniques, along with technological advancement in tandem mass spectrometry has been employed as a powerful tool to study protein phosphorylation and its biological relevance (Chen and White, 2004). In this review, a variety of separation and enrichment methods for phosphoproteins and phosphopeptides, their features as well as applications in phosphoproteomics research are described.

## Phosphoproteomics technologies

Low stoichiometry of phosphorylated proteins and low ionization efficiency of phosphopeptide are two major challenges for protein phosphorylation detection. To reduce sample complexity, it is necessary to enrich the modified proteins and/or peptides before mass spectrometry (MS) analysis. Commonly used enrichment techniques were summarized in Table 1, with enrichment at the peptide level as a popular strategy. A successful phosphoproteomics study depends not only on the selective enrichment of phosphopeptides, but also on accurate detection and quantitation of the peptides, as well as precise mapping of the phosphorylation sites. Advances in these areas have been extensively reviewed (Batalha et al., 2012; Fíla and Honys, 2012; Kline and De Luca, 2014; Silva-Sanchez et al., 2015). Most of the technologies were developed in animal and yeast systems, and subsequently applied in plants. Here we briefly describe the advancement of phosphoproteomics technologies in plant research (Table 2).

**Table 1:** **Phosphopeptide/phosphotprotein enrichment methodologies**.

**Enrichment method**	**Description**	**Advantage**	**Disadvantage**	**References**
Immunoaffinity enrichment	Use of antibodies directed against pTyr, pSer, pThr, and more recently against the surrounding consensus sequences for pSer/pThr.	Highly specific.	Low efficiency, high cost, use of different antibodies for different phosphorylation motifs.	Stokes et al., 2012
Immobilized metal affinity chromatography (IMAC)	Negatively charged phosphate groups on the phosphorylated amino acids interact with positively charged metal ions such as Ni^2+^, Fe^3+^, Ga^3+^, Zr^4+^, and Ti^4+^ that are chelated with silica or agarose through nitriloacetic acid or iminodiacetic acid.	Good for both phosphoproteins and phosphopeptides. When used with peptides, it can enrich mono- and multiple phosphorylated peptides.	Tends to bind strongly to monophosphorylated peptides, which makes it difficult for elution. Non-specific binding of acidic peptides can occur.	Fíla and Honys, 2012
Metal oxide affinity chromatography (MOAC)	Similar to IMAC, the phosphate groups on the amino acids interact with positively charged metal oxides, e.g., titanium or zirconium that acts as anchoring molecules to trap phosphopeptides through the formation of multi-dentate bonds.	Good for both phosphoproteins and phosphopeptides. When used with peptides, it can enrich mono- and multiple phosphorylated peptides.	Tends to binds strongly to multiple phosphorylated peptides, which makes it difficult for elution. Nonspecific binding of acidic peptides can occur.	Gates et al., 2010
Phos-Tag chromatography,	Uses 1,3-bis[bis(pyridine-2-ylmethyl)amino]propan-2-olato dizinc(II) complex as a selective phosphate binding tag in aqueous solution at neutral pH.	Increased sensitivity due to complete deprotonation of phosphoproteins/ phosphopeptides at neutral pH. Elution at the physiological pH allow for protein activity and functional analysis.	Mainly used to confirm the phosphorylation state in relatively pure proteins, but not with complex mixtures.	Kinoshita et al., 2006
Prefractionation by strong cation exchange (SCX) and strong anion exchange (SAX)	In SCX, tryptic peptides often carry a charge of +2, except for phosphopetides with a net charge of +1, making them elute early in the chromatography. SAX retains phosphor-peptides, allowing separation based on the number of phosphorylated residues.	Used for fractionation of highly complex mixtures, it can be performed on-line with mass spectrometry.	Similar degree of unspecific binding as IMAC and MOAC.	Leitner et al., 2011
Hydrophilic interaction liquid chromatography (HILIC)	Phosphopeptides with polar phosphate groups are strongly retained on the HILIC stationary phase resulting in separation from non-phosphorylated species.	Good for both phosphoproteins and phosphopeptides. When used with peptides, it can enrich mono- and multiple phosphorylated peptides.	Similar degree of unspecific binding as IMAC and MOAC.	(Yang et al., 2013)
Electrostatic repulsion hydrophilic interaction chromatography (ERLIC)	ERLIC is a variation of HILIC using electrostatic repulsion as an additional phase to adjust selectivity by varying pH or organic solvents.	Good for both phosphoproteins and phosphopeptides. When used with peptides, it can enrich mono- and multiple phosphorylated peptides.	Similar degree of unspecific binding as IMAC and MOAC.	Gan et al., 2008
Hydroxyapatite chromatography	It takes advantage of the strong interaction between positively charged hydroxyapatite and phosphate ions.	Good for fractionating mono-, di-, tri-, and multi-phosphorylated peptides when using gradient of a phosphate buffer.	Developed with phosphoprotein standards, not tested with complex samples.	Mamone et al., 2010

**Table 2:** **Representative plant phosphoproteomics work in the past decade**.

**Plant materials**	**Phosphopeptides/phosphoproteins**	**Enrichment method**	**Quantitation method**	**Phosphorylationsite mapping**	**MS instrument**	**References**
Arabidopsis plasma membrane	283 phosphopeptides	IMAC	None	Mascot	QTOF Ultima (Waters)	Nuhse et al., 2004
Arabidopsis leaves	317 phosphopeptides	Phospho- protein kit	iTRAQ	Mascot	QTRAP (AB Sciex)	Jones et al., 2006
Arabidopsis leaves	16 phosphopeptides	None	MRM	MS^3^ de novo	TSQ Quantum (Thermo)	Glinski and Weckwerth, 2005
Arabidopsis suspension cells	1168 phosphopeptides	TiO_2_	SILAC	MSQuant	LTQ FT-ICR (Thermo)	Benschop et al., 2007
Arabidopsis leaves	111 phosphoproteins	Pro-Q Diamond	2-DE	Mascot	QSTAR XL (AB Sciex)	Shin et al., 2007
Arabidopsis plasma membrane	67 phosphopeptides	IMAC	Precursor ion intensity	MSQuant	LTQ (Thermo)	Niittylä et al., 2007
Tomato leaves	48 proteins	TiO_2_	Precursor ion intensity	VEMS	QTOF (Micromass)	Stulemeijer et al., 2009
Arabidopsis leaves	3589 phosphopeptides	TiO_2_ and FeCl_3_	Spectral counting	Mascot	Orbitrap (Thermo)	Reiland et al., 2011
Arabidopsis leaves	3 phosphopeptides	None	MRM	Previously determined	TSQ Quantum (Thermo)	Schulze et al., 2012
Arabidopsis leaves	5386 phosphopeptides	PolyMAC	Precursor ion intensity	PhosphoRS	Orbitrap Velos (Thermo)	Wang et al., 2013b
Arabidopsis leaves	1 phosphopeptide	None	MRM	Previously determined	4000 QTRAP (AB Sciex)	Prado et al., 2013
Wheat leaves	2305 phosphopeptides	TiO_2_ and HILIC	TMT	Mascot	Orbitrap Velos (Thermo)	(Yang et al., 2013)
Arabidopsis microsome	1229 phosphopeptides	TiO_2_	SILAC	Mascot	Orbitrap XL (Thermo)	Stecker et al., 2014
Cotton leaves	1315 phosphopeptides	TiO_2_	iTRAQ	PhosphoRS	Q Exactive (Thermo)	Fan et al., 2014
Arabidopsis leaves	14 phosphopeptides	TiO_2_	MRM	Mascot	QTRAP 5500 (AB Sciex)	Minkoff et al., 2015

*LTQ, linear ion trap; VEMS, Virtual Expert Mass Spectrometrist; MRM, multiple reaction monitoring; TOF, time of flight; FT-ICR, Fourier transform ion cyclotron resonance. Please refer to the text for other abbreviations*.

### Enrichment strategies

The most widely used enrichment method for phosphopeptides takes advantage of the affinity binding between negatively charged phosphate and positively charge metal ions (Fíla and Honys, 2012). Immobilized metal affinity chromatography (IMAC) is often coupled with strong cation exchange (SCX) for two-step phosphopeptide enrichment. For example, in a SCX-IMAC experiment, three times more phosphopeptides were identified when compared to the use of SCX or IMAC alone (Trinidad et al., 2006). The first reported SCX-IMAC application in plants resulted in identification of 283 phosphopeptides (Nuhse et al., 2004). In addition, Polymer-based Metal-ion Affinity Capture (PolyMAC) is a variant of IMAC, also showed high selectivity. For instance, employment of complementary PolyMAC-Titanium (Ti) and PolyMAC-Zirconium (Zr) ion affinity chromatography lead to identification of 5386 unique phosphopeptides (Wang et al., 2013a).

Metal dioxide especially titanium dioxides (TiO_2_) and zirconium dioxides (ZrO_2_) are gaining popularity for phosphopeptide enrichment. A comparison of the performance of TiO_2_ and ZrO_2_ performed with α-casein and β-casein as standard proteins showed that TiO_2_ tends to enrich multiply phosphorylated peptides, and ZrO_2_ tends to enrich singly phosphorylated peptides. A serial enrichment procedure with both TiO_2_ and ZrO_2_ can significantly increase the efficiency of capturing phosphopeptides in biological samples (Kweon and Håkansson, 2006; Gates et al., 2010). Metal dioxide enrichment could also be coupled with other peptide fractionation methods. For instance, a combination of TiO_2_ enrichment and hydrophilic interaction liquid chromatography (HILIC) resulted in identification of 2305 phosphopeptides belonging to 964 proteins in wheat (Yang et al., 2013). Electrostatic repulsion hydrophilic interaction chromatography (ERLIC), a variation of HILIC that uses electrostatic repulsion as an additional chromatography stationary phase, had also been used successfully for selectively enrichment of phosphopeptides (Gan et al., 2008; Loroch et al., 2013).

### Quantitative phosphoproteomics

Quantitative phosphoproteomics is aimed to enable a better understanding of phosphorylation regulated biological events. Comparative phosphoproteomics of wild-type and mutant plants or control and treated plants could be conducted in many ways. In general, the approaches can be grouped into gel-based, gel-free, stable isotope labeling, or label-free. Two-dimensional gel electrophoresis (2-DE) has been a widely used technology that resolves thousands of proteins by isoelectric point and molecular weight. Pro-Q Diamond is a fluorescent stain that provides a convenient method for selectively staining phosphoproteins in acrylamide gels. The result shows a global map of the modified proteins and their relative abundances compared to non-phosphorylated counterparts when a total protein staining is used after Pro-Q Diamond staining. Differentially phosphorylated proteins in wild-type and *snk2.8* mutant Arabidopsis plants were analyzed using 2-DE and Pro-Q, and putative substrates of SnRK2.8 were identified (Shin et al., 2007).

Stable isotope labeling has been applied in plant phosphoproteomics successfully using a gel-free approach, for example, stable isotope labeling of amino acids in cell culture (SILAC). The first SILAC in plants was done by introducing ^15^N in Arabidopsis suspension cells (Benschop et al., 2007) (Table 2). The methodology has been improved over the years and found more applications (Schütz et al., 2011; Stecker et al., 2014). Another labeling approach introduces multiplex isobaric tags to isolated proteins or digested peptides *in vitro*. Commonly used tags include isobaric tags for relative and absolute quantification (iTRAQ) and tandem mass tags (TMT). The tags are designed to be isobaric during MS and fragment to reveal differential low mass ion reporters during MS/MS. Due to its capability of multiplexing up to 10 samples in a single experiment and the enrichment effect for low abundance proteins, iTRAQ/TMT labeling has become popular in plant phosphoproteomics (Jones et al., 2006; Yang et al., 2013; Fan et al., 2014).

While both *in vivo* and *in vitro* label methods are limited by the number of samples, label free approaches enable quantitative phosphoproteomics of unlimited number of samples. There are two main methods in label free quantitation. The first is based on precursor ion peak intensity/area, and the second is based on the number of MS/MS spectra acquired for a given peptide (known as spectral counting). Both methods were used in plant phosphoproteomics (Reiland et al., 2011; Engelsberger and Schulze, 2012; Wang et al., 2013a). For instance, Reiland et al. (2011) characterized the function of a thylakoid-associated kinase STN8 in the fine-tuning of cyclic electron flow, which is regulated by the phosphorylation/dephosphorylation event.

In addition to these large scale discovery phosphoproteomics approaches, multiple reaction monitoring (MRM) has been used for quantification of targeted phosphopeptides (Glinski and Weckwerth, 2006; Schulze et al., 2012; Minkoff et al., 2015). A triple quadrupole is typically used for the MRM measurement, in which the first quadrupole (Q1) is set as a filter for the precursor ion with predetermined mass and Q3 is set to measure a specific fragment ion. The specific combination between a precursor ion and a fragment ion is called a transition and multiple transitions can be used to determine the relative and absolute (with synthesized peptide standards) levels of phosphopeptides (Schulze et al., 2012).

## Applications of phosphoproteomics in plant biology research

### Phosphoproteomics of signal transduction

Protein phosphorylation in signal transduction is an important area of current plant biology research. Many key proteins such as kinases, transcription factors, and ubiquitin ligases function through reversible protein phosphorylation in the signal transduction cascade (Hunter, 2000). In recent years, it has become apparent that analysis of signaling networks is required for the understanding of the dynamic and complex mechanisms underlying cellular functions and outputs. Most of the studies in plants have often been focused on protein kinases and identification of the phosphorylated substrates.

**Figure 1:**
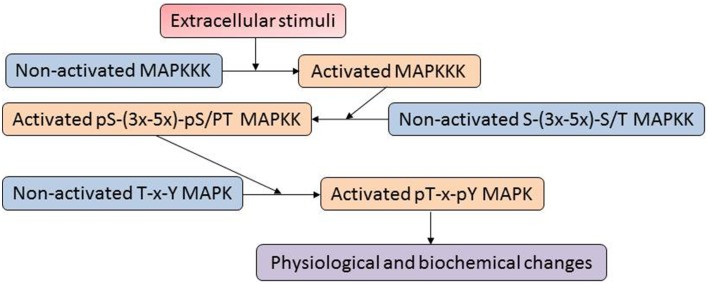
**A typical mitogen-activated proteins (MAP) kinase cascade**. The MAPK cascades are generally organized as modular pathways, in which the activation of upstream MAPKKKs leads to the sequential phosphorylation and subsequent activation of downstream MAPKKs and MAPKs.

The mitogen-activated protein kinases (MAPKs) constitute one of the most important signaling mechanisms in plants, and they play essential roles in linking the perception of different stimuli with cellular adaptive responses. The MAPK signal transduction pathways are evolutionarily conserved in all eukaryotic organisms such as plants, yeast, fungi, insects, nematodes, and mammals (Mishra et al., 2006). A MAPK cascade is minimally composed of distinct combinations of at least three protein kinases: a MAPKKK (MAP3K), a MAPKK (MAP2K) and a MAPK, which activate in a sequential manner via phosphorylation (Figure 1). An activated MAPKKK firstly phosphorylates two serine and/or threonine residues (S/T-X3-5-S/T) located within the activation loop of the MAPKK. Activated MAPKKs in turn trigger MAPK activation through dual phosphorylation of a highly conserved T-X-Y motif in the activation loop of MAPKs (Hamel et al., 2012). In a proteomic analysis of plasma membrane isolated from maize roots, four isoforms of Pto-interacting-like kinase 1 (PTI1) showed increased levels in response to low and high iron conditions (Hopff et al., 2013). Interestingly, a previous oxidative stress study in Arabidopsis demonstrated that interaction of a PTI1-like kinase (PTI1-4) with another serine/threonine protein kinase, oxidative signal-inducible 1 (OX1), mediates oxidative stress signaling. In addition, PTI1-4 was found to interact with MPK6 in the same protein complex (Forzani et al., 2011). These results imply that the PTI signals may function through the OXI1 and MPK6 signaling cascades. Recently, Hoehenwarter et al. (2013) developed a two-step chromatography combining phosphoprotein enrichment using Al(OH)_3_-based metal oxide affinity chromatography with phosphopeptide enrichment using TiO_2_-based metal oxide affinity chromatography to enrich phosphopeptides from complex *A. thaliana* protein samples. The method was successfully applied to identify MAPK substrates. A large number of novel phosphorylation sites and 141 MAPK substrate candidates (mostly novel) have been identified. For example, time for coffee (TIC) and non-phototropic hypocotyl 3 (NPH3), which are involved in circadian clock and phototropism, were found to be MPK3/6 substrates. The result suggests that plant circadian rhythm and phototropism may be regulated by the MAPK signaling network.

Abscisic acid (ABA) is a phytohormone that plays an important role in many aspects of plant life. For example, ABA is essential for regulating seed maturation and stomatal closure under abiotic and biotic stresses (Hubbard et al., 2010). Protein phosphorylation and dephosphorylation play a central role in ABA signaling. Multiple signaling components have been found to undergo phosphorylation/dephosphorylation regulation to control stomatal movement in response to ABA (Zhang et al., 2014). A simplified ABA signaling model consists of the soluble ABA receptors (members of the pyrabactin resistance 1 (PYR1) and PYR1-like (PYL) proteins, also known as regulatory component of ABA receptor (RCAR) family, and collectively referred to as PYR/PYL/RCAR), a subgroup of type 2C protein phosphatases (PP2Cs), and the SNF1-related protein kinase 2 (SnRK2) family (Umezawa et al., 2010). Umezawa et al. (2013) studied protein phosphorylation networks in ABA signaling using phosphoproteomics of Arabidopsis treated with ABA and dehydration stress, as well as *snrk2* mutants to identify SnRK2-dependent protein components. Comparative analysis between ABA treatment and dehydration stress revealed that dehydration stress induced multiple protein phosphorylation pathways in addition to the ABA-dependent pathway, supporting that multiple protein kinases are involved in dehydration stress signaling, including SnRK2s, MAPKs, and calcium-dependent protein kinases (CDPKs) (Umezawa et al., 2013). Further studies will be required for understanding how multiple kinases mediate dehydration stress signaling. It appeared that subclass III SnRK2s may be uniquely employed during ABA responses, and subclass II SnRK2s are the main subclass that regulates dehydration stress responses, although they are also activated by ABA. By integration of genetics with phosphoproteomics, it is possible to connect protein kinases with their *in vivo* signaling pathways. In particular, this study provided insights into the ABA signaling pathway by identifying potential substrate proteins of SnRK2s (Umezawa et al., 2013).

### Phosphoproteomics of subcellular compartments

Phosphoproteomics studies were often performed in a shotgun fashion, with the identification of hundreds and thousands of proteins that lead to a very complicated set of phosphoproteins across subcellular compartments and organelles (Table 2), leading to a poor understanding of the networks that regulate the cellular activities (Jung et al., 2000). Compartmentalization in eukaryotes offers a practical approach to study subcellular phosphoproteomics networks, with a reduced population of identified proteins. There are about 3000 proteins in the chloroplasts of Arabidopsis, but only four kinases were previously identified. It may be feasible to find specialized kinases or families of kinases that can potentially show differential activities in the chloroplasts (Millar and Taylor, 2014; van Wijk et al., 2014). A meta-analysis of 27 publications of phosphoproteomics data sets in Arabidopsis comprises 60,366 phosphopeptides matched to 8141 non-redundant proteins. The phosphoproteins showed predicted subcellular distribution in the following categories: nucleus, secretory (containing endoplasmic reticulum, Golgi, plasma membrane, cell wall, and vacuolar), cytosol, other/unknown, intra-plastid, mitochondria, and peroxisome (van Wijk et al., 2014). The study of phosphoprotein compartmentalization supports the hypothesis that a fine mechanism helps to maintain and regulate protein translation, post-translational metabolism, signaling, and trafficking through the cells (Millar and Taylor, 2014). Some studies have already started to focus on PTMs in subcellular compartments and here we describe a few examples.

Jones et al. (2009) performed a phosphoproteomic analysis of the nuclei-enriched fractions prepared from suspension cell cultures and seedlings of *A. thaliana*. The work led to the identification of 416 phosphopeptides from 345 proteins. Two thirds of the proteins are known or predicted to be nuclear localized, and one half of the nuclear localized proteins have novel phosphorylation sites. Many phosphorylation sites and kinase motifs were identified on proteins involved in nuclear transport (e.g., Ran-associated proteins), and on transcription factors, chromatin remodeling proteins, and spliceosome components. Surprisingly, many novel phosphopeptides from proteins involved in vesicle trafficking such as components of the exocyst complex (SEC10, SEC51, and SEC5a-like) were identified. How phosphorylation of these SEC proteins plays a role in vesicle trafficking is intriguing. Recently, phosphorylation of Sec31 by a casein kinase 2 was found to control the duration of COPII vesicle formation, decrease its association with ER and promote ER-to-Golgi trafficking (Koreishi et al., 2013).

Subcellular proteomics can address conserved mechanisms underlying plant responses to stresses. By analyzing the phosphorylation changes in proteins of microsomal fractions from *A. thaliana* and *Oryza sativa*, Chang et al. (2012) found similar phosphoproteins between the species including photosystem II reaction center protein H PsbH. Both Arabidopsis and rice showed an increased ratio for a diphosphorylated peptide (ApTQpTVEDSSRSGPR) of PsbH as a response to salt stress. Interestingly, the two phosphorylation sites (Thr2 and Thr4) are found to be evolutionarily conserved in many plants using sequence alignment.

Light plays a crucial role in the regulation of protein phosphorylation in photosynthetic thylakoid membranes. In Arabidopsis, the thylakoid Ser/Thr protein kinase 7 (STN7) and STN8 kinases are light regulated and participate in phosphorylation of thylakoid membrane proteins and stroma proteins. Ingelsson and Vener (2012) performed a thylakoid phosphoproteomics study using Arabidopsis wild-type and the *STN* mutants *stn7, stn8*, and *stn7stn8*. The results showed that STN7 is required for the phosphorylation of pTAC16 at the Thr451, and pTAC16 was found to be distributed between thylakoids and nucleoid. In addition, the results suggest that pTAC16 could anchor DNA to the thylakoid membrane, and it was proposed that STN7-dependent phosphorylation of pTAC16 may regulate membrane-anchoring functions of the nucleoid.

### Phosphoproteomics of plant growth and development

Sucrose non-fermenting 1 related kinase (SnRK1) acts as a sensor of energy levels in plant development, and regulates plant growth by maintaining energy homeostasis during stress conditions (Tsai and Gazzarrini, 2014). It is activated by sugar depletion, energy depletion in the dark and hypoxia (Baena-González and Sheen, 2008). Trehalose 6-phosphate (T6P) is a signaling molecule involved in the regulation of embryonic and vegetative development, flowering time, and meristem determinacy. An increase in the levels of T6P led to metabolic changes that promote plant growth. However, T6P regulates SnRK1 by inhibiting its activity. SnRK1 and T6P are global regulatory molecules that also interact with plant hormones, and along with ABA modulate several crucial cellular activities such as seed maturation and germination, ABA sensitivity and signaling, vegetable growth, and flowering regulation (Tsai and Gazzarrini, 2014). Seed germination is known to be controlled by phytohormones, including gibberellins (GAs) and ABA, which play antagonistic roles as positive and negative regulators, respectively (Seo et al., 2006). Many protein kinases and phosphatases participate in ABA signaling to regulate seed germination. Recently, Han et al. (2014) used PolyMAC phosphopeptide enrichment and gel-free proteomics identified a total of 933 phosphorylated peptides corresponding to 413 proteins in rice embryos during early stages of germination. By quantitative normalization of phosphoprotein abundance and One-Way ANOVA testing, 149 phosphorylated proteins were found to be significantly changed in abundance during germination. Among the phosphoproteins, seven brassinosteroid (BR) signaling pathway-related proteins were identified and three (BR signaling kinase 1, BR-insensitive 2, and BR-insensitive 1 suppressor 1) showed significant increases in phosphoprotein abundance during the early stages of germination. In addition, treatment with brassinolide promoted the rice seed germination. These results suggest that brassinosteroid signal transduction plays an important role in triggering seed germination.

Plant vegetative growth is important for biomass accumulation and potential biofuel applications. A recent phosphoproteomic study of *Brachypodium distachyon* as a model biofuel plant using TiO_2_ enrichment and LC-MS/MS has identified a total of 1470 phosphorylation sites in 950 phosphoproteins (Lv et al., 2014). Among the phosphoproteins, there were 58 transcription factors, 84 protein kinases, 8 protein phosphatases, and 6 cellulose synthases. Through bioinformatic analysis, a protein kinase and phosphatase centered network related to rapid vegetative growth was deciphered. For example, a MAPK signaling cascade might play an important role in leaf growth and development (Lv et al., 2014). This finding is very interesting, considering MAPK cascade is generally involved in plant stress responses (Mishra et al., 2006).

## Identification and functional analysis of novel phosphorylation sites

The identification of protein phosphorylation sites has been difficult in the past. Nowadays, high throughput modern technologies such as tandem MS have promoted large-scale discoveries of new phosphorylation sites and phosphoproteins in recent years. Rao and Møller (2012) initiated a large-scale study of phosphorylation site occupancy in eukaryotic proteins. They analyzed the occurrence and occupancy of phosphorylation sites in a large number of eukaryotic proteins, and provided insights into protein phosphorylation and related processes. Phosphorylation probability was found to be much higher in both termini of protein sequences (much more in the C-terminus) than middle parts of the sequences. A large proportion (51.3%) of the occupied sites had a nearby phosphorylation within a distance of 10 amino acid residues. This proportion is very high compared to the expected value of 16.9%. More than half of the phosphorylated sites fall within a small number of motifs.

A large phosphoprotein, the RNA surveillance protein UP-frameshift 1 (Upf1) in *Saccharomyces cerevisiae*, has only been partially characterized for phosphorylation sites, but the functional relevance of the phosphorylation has not been studied. Lasalde et al. (2014) used tandem MS and *in vitro* phosphorylation assays to identify novel phosphorylation sites in UPF1. A total of 11 phosphorylated residues of UPF1 were identified. Sequence alignment of UFP1 from lower and higher eukaryotes showed complete conservation of the phosphorylated residue Y-754. Residues corresponding to *S. cerevisiae* UPF1 T-194, S-492, Y-738, and S-748 were similar to those in the homologs of *Homo sapiens, Mus musculus, Drosophila melanogaster*, and *A. thaliana*. Since the phosphorylated residues in UPF1 were clustered in four small regions, each one was tested to determine its importance by independently deleting the four individual regions. The deletion mutant lacking phospho-motif-4 was not able to complement the Nonsense-Mediated mRNA Decay (NMD) defect as revealed by Northern blot analysis. To test the role of phospho-motif-4 in translation termination efficiency, a well-established dual luciferase assay was used. The deletion-mutant lacking phospho-motif-4 was not able to rescue this defect, indicating that this motif has a role in translation termination efficiency. To dissect the sequences within phospho-motif-4 required for NMD activity, PCR-mediated mutagenesis was used to generate three additional deletion mutants (736–745, 746–750, 751–751). The results revealed that deletion of residues 736–745 reduced NMD activity as measured by Northern blot analysis. To test the functional role of Y738 and Y742, site-directed mutagenesis was used to create phosphorylation mimic mutants. Interestingly, the Y738F and Y742F fully rescued NMD activity of a chromosomal UPF1 deletion-mutant strain, indicating that they are not compromised in their ability to function in NMD. These results provided strong evidence that UPF1's ability to promote translational termination fidelity is depended on the conserved C-terminal phosphorylation motif, which is important for its NMD activity.

## Concluding remarks

Phosphopeptide enrichment and MS have been essential tools for studying protein phosphorylation. It is challenging to directly detect phosphoproteins in biological samples due to the low abundance and low stoichiometry of phosphorylation in different biological processes. The enrichment methods of phosphoproteins/phosphopeptides from complex mixtures have greatly improved over the years. For instance, combining the titanium (Ti^4+^)-based IMAC and the reverse phase (RP)-strong cation exchange (RP-SCX) biphasic trap column-based online RPLC is a great example of the advancements (Bian et al., 2012; Wang et al., 2013b). The recent development of specific labeling techniques has greatly aided the quantification of phosphorylation profiles and their stress-induced changes. Especially, iTRAQ and TMT *in vitro* labeling and SILAC *in vivo* labeling have shown to be successful in combination with IMAC and MS (Isner et al., 2012; Yang et al., 2013; Zhang et al., 2013; Stecker et al., 2014). These studies have revealed novel nodes and edges in signaling pathways and regulatory processes that are dependent on phosphorylation. Despite many new insights gained from quantitative phosphoproteomics, improvements are required to enable a comprehensive description of total and PTM proteomes. Currently, LC-MS/MS based phosphoproteomic technologies have established as an indispensable tool in identification of novel phosphorylation sites and signaling pathways. As large data sets accumulate, informatics tools will be indispensable, e.g., informatics has revealed phosphorylation probability to be frequent at the termini of protein sequences. Taken together, researchers have provided not only new insights into the complex phosphorylation regulatory networks in plants, but also important resources for future functional studies of protein phosphorylation in plant growth and development.

### Conflict of interest statement

The authors declare that the research was conducted in the absence of any commercial or financial relationships that could be construed as a potential conflict of interest.
